# Evaluation the kill rate and mutant selection window of danofloxacin against *Actinobacillus pleuropneumoniae* in a peristaltic pump model

**DOI:** 10.1186/s12917-024-04016-9

**Published:** 2024-06-03

**Authors:** Hongjuan Wang, Chengshui Liao, Ke Ding, Longfei Zhang, Lei Wang

**Affiliations:** 1 College of Animal Science and Veterinary Medicine, Henan Institute of Science and Technology, Xinxiang, 453003 China; 2 Institute of Traditional Chinese Veterinary Medicine, College of Veterinary Medicine, Gansu Agricultural University, Lanzhou, 730070 China; 3 The Key Lab of Veterinary Biological Products, Henan University of Science and Technology, Luoyang, 471000 China; 4 Laboratory of Functional Microbiology and Animal Health, Henan University of Science and Technology, Luoyang, 471023 China; 5 Institute of Farmland Irrigation, Chinese Academy of Agricultural Sciences, Xinxiang, 453003 China

**Keywords:** *Actinobacillus pleuropneumoniae*, Kill rate, Mutation selection window, Peristaltic pump model, PK/PD integration

## Abstract

**Background:**

*Actinobacillus pleuropneumoniae* is a serious pathogen in pigs. The abundant application of antibiotics has resulted in the gradual emergence of drugresistant bacteria, which has seriously affected treatment of disease. To aid measures to prevent the emergence and spread of drug-resistant bacteria, herein, the kill rate and mutant selection window (MSW) of danofloxacin (DAN) against *A. pleuropneumoniae* were evaluated.

**Methods:**

For the kill rate study, the minimum inhibitory concentration (MIC) was tested using the micro dilution broth method and time-killing curves of DAN against *A. pleuropneumoniae* grown in tryptic soy broth (TSB) at a series drug concentrations (from 0 to 64 MIC) were constructed. The relationships between the kill rate and drug concentrations were analyzed using a Sigmoid E_max_ model during different time periods. For the MSW study, the MIC_99_ (the lowest concentration that inhibited the growth of the bacteria by ≥ 99%) and mutant prevention concentration (MPC) of DAN against *A. pleuropneumoniae* were measured using the agar plate method. Then, a peristaltic pump infection model was established to simulate the dynamic changes of DAN concentrations in pig lungs. The changes in number and sensitivity of *A. pleuropneumoniae* were measured. The relationships between pharmacokinetic/pharmacodynamic parameters and the antibacterial effect were analyzed using the Sigmoid E_max_ model.

**Results:**

In kill rate study, the MIC of DAN against *A. pleuropneumoniae* was 0.016 µg/mL. According to the kill rate, DAN exhibited concentration-dependent antibacterial activity against *A. pleuropneumoniae*. A bactericidal effect was observed when the DAN concentration reached 4–8 MIC. The kill rate increased constantly with the increase in DAN concentration, with a maximum value of 3.23 Log_10_ colony forming units (CFU)/mL/h during the 0–1 h period. When the drug concentration was in the middle part of the MSW, drugresistant bacteria might be induced. Therefore, the dosage should be avoided to produce a mean value of AUC_24h_/MIC_99_ (between 31.29 and 62.59 h. The values of AUC_24h_/MIC_99_ to achieve bacteriostatic, bactericidal, and eradication effects were 9.46, 25.14, and > 62.59 h, respectively.

**Conclusion:**

These kill rate and MSW results will provide valuable guidance for the use of DAN to treat *A. pleuropneumoniae* infections.

## Background

Porcine contagious pleuropneumonia is a serious respiratory disease of pigs caused by *Actinobacillus pleuropneumoniae*, which mainly grows in the porcine nasal cavity, tonsil recess, and alveolar cells [1–3]. The clinical symptoms mainly include acute and chronic symptoms. The acute symptoms are fibrinous hemorrhagic pneumonia and necrotic pneumonia, which often cause pig death. Chronic disease causes a decline in the pig’s feed intake, which slows down pig weight gain and seriously affects production in the pig breeding industry [4]. To date, the disease has spread to all regions of the world, seriously affecting pig production and causing huge economic losses to the breeding industry, consequently attracting increased research attention [5–7]. However, there are more than 19 serovars of *A. pleuropneumoniae*; therefore, the protective effect of the currently available vaccine is not satisfactory [8]. Consequently, antimicrobials still play an important role in the prevention and treatment of this disease, including cephalosporins, fluoroquinolones, and macrolides [9–12].

Fluoroquinolones are concentration-dependent drugs and have been widely applied to treat gram-negative bacterial infections in humans and animals [13, 14]. However, the abundant use of fluoroquinolones has resulted in the emergence of drug resistant bacteria worldwide, which seriously threatens public health. Therefore, dosage regimens should be optimized to prevent the emergence of drug-resistant bacteria. Pharmacokinetic/pharmacodynamic (PK/PD) synchronization modeling is an important method of dosage optimization [15, 16]. Especially, the mutation selection window (MSW)-based PK/PD model significantly supports methods to inhibit the production and transmission of drug resistant bacteria by analyzing the relationship between PK/PD parameters based on the minimum inhibitory concentration (MIC) or mutant prevention concentration (MPC) [17–19]. To date, MSW-based PK/PD analysis has been carried out for several kinds of antibacterial agents, among which fluoroquinolones are the most suitable drugs [20–23]. The resistance mechanism of bacteria against fluoroquinolones usually emerges gradually from gene point mutations, which is mostly consistent with the mutation mechanism in MSW theory [24, 25]. Danofloxacin (DAN) is a third generation fluoroquinolone antibacterial that is only used in animals. It has good antibacterial activity against bacteria and mycoplasmas, and has been applied to treat porcine respiratory diseases caused by *A. pleuropneumoniae*, *Pasteurella multocida*, and *Mycoplasma hyopneumoniae* [26]. In a previous study, we carried out MSW analysis of DAN against *A. pleuropneumoniae* in tissue cage fluid (TCF) [12]. However, the PK parameters of DAN in TCF are obviously different from those in the lung, and there are many difficulties in establishing a lung infection model for PK/PD studies. Hence, it is important to establish a peristaltic pump infection model to simulate lung infection.

Therefore, to clarify the antibacterial activity of DAN against *A. pleuropneumoniae* and prevent the emergence of resistant bacteria, we carried out a PK/PD study based on the kill rate and MSW of DAN against *A. pleuropneumoniae*. Firstly, the in vitro kill rate of DAN against *A. pleuropneumoniae* was assessed to study its antibacterial characteristics. Then, an in vitro peristaltic pump infection model was established according to the PK characteristics of DAN in pig lungs, which was used to study the MSW of DAN against *A. pleuropneumoniae*. We believe that the results of the present study can precisely clarify the antibacterial activities of DAN against *A. pleuropneumoniae* and will provide a valuable guide for dosage regimen designation to prevent the emergence of resistant bacteria.

## Materials and methods

### Strains, reagents, and the peristaltic pump model

The standard strain of *A. pleuropneumoniae*, CVCC259, serovar 1, was provided by the Chinese Veterinary Culture Collection Center (Qingdao, China). DAN mesylate powder (99%) was provided by Guangdong Dahuanong Biotechnology Company (Yunfu, China). Tryptic soy broth (TSB), Mueller-Hinton agar (MHA), nicotinamide adenine dinucleotide (NAD), and newborn bovine serum were provided by Guangdong Huankai Microbiology Technology (Shanghai, China). The peristaltic pump (BT100-1 F), pump head (DG-2-B/D, 10 roller), and rubber hose (inner diameter ≤ 3.17 mm, wall thickness 0.8–1 mm) were purchased from Baoding Longer Constant Pump Ltd. (Baoding, China). Fiber dialysis tubes (Float-A-Lyzer, 1000 KDa, 10 mL) were purchased from SpectrumLabs Inc (California, US). The TSB and MHA used to culture *A. pleuropneumoniae* contained 4% newborn bovine serum and 1% NAD (1 mg/mL).

### MIC and kill curves of DAN against *A. Pleuropneumoniae* in TSB

A micro-broth dilution method was applied to test the MIC of DAN against *A. pleuropneumoniae* according to the Clinical & Laboratory Standards Institute (CLSI) standards [27]. In detail, 100 µL of DAN-containing TSB and 100 µL of log phased *A. pleuropneumoniae* were added to one to nine wells of a 96-well plate, such that the final concentration of DAN ranged from 0.004 to 1 µg/mL and the *A. pleuropneumoniae* concentration was 10^5^ colony forming units (CFU)/mL. Both positive and negative controls were set. The plate was then incubated in a thermostatic incubator (5% CO_2_, 37 ℃) for 18–20 h. The MIC was determined as the minimum drug concentration that inhibited bacterial growth. The experiment was carried out in triplicate.

For the time-killing curves, eight drug concentration groups were created by twofold dilution (0.5 to 64 MIC) and a control group (0 MIC) wa set. Briefly, 100 µL of DAN solution (a 100 times dilution of the initial concentration), 1 mL log phase *A. pleuropneumoniae* (10^7^ CFU/mL), and 8.9 mL TSB were added into 15 mL centrifuge tubes and cultured in an incubator (37 ℃, 5% CO_2_). The population of *A. pleuropneumoniae* was counted by the agar plate method at 0, 1, 3, 6, 9, 12, and 24 h, respectively. The detection limit of *A. pleuropneumoniae* was 50 CFU/mL. When the number of *A. pleuropneumoniae* was lower than 50 CFU/mL, 50 CFU/mL was applied to construct the kill curves. All experiments were carried out in triplicate. On the kill curves, the vertical axis was the mean number (Log_10_ CFU/mL) of *A. pleuropneumoniae* and the horizontal axis was the culture time.

### Analysis of the kill rate and DAN concentration

The kill rate (Log_10_ CFU/mL/h) was measured as the slope of the kill curve during each time period. In this study, we only analyzed the kill rate within 0–3 h (0–1 h, 1–3 h, and 0–3 h) because *A. pleuropneumoniae* could not be detected after 3 h at 32 and 64 MIC.

A Sigmoid E_max_ model was applied to analyze the relationships between kill rates and DAN concentrations using WinNonlin software (version 5.2.1, Pharsight, Mountain View, CA, USA). The model formula is described as follows:

Formula 1 $$E = {E_0} + \frac{{\left( {{E_{\max }} - {E_0}} \right) \times C_e^N}}{{C_e^N + EC_{50}^N}}$$

where E is the kill rate; E_max_ is the maximum kill rate of DAN during each time period; E_0_ is the kill rate in blank TSB; C_e_ is the DAN concentration; N is the Hill coefficient, representing the steepness of the kill curve; and EC_50_ is the DAN concentration to produce a 50% maximum kill rate.

The R^2^ value represented the fit degree of the kill rate and drug concentration at each time period. The larger the R^2^ value, the higher the correlation between the kill rate and the DAN concentration.

### Determination of MIC, MIC_99_, and MPC of DAN against *A. Pleuropneumoniae* in MHA

The methods to determine the MIC, MIC_99_, (the lowest concentration that inhibited the growth of the bacteria by ≥ 99%) and MPC were performed according to our previously published methods using the agar plate method [12]. The details of the methods were as follows:

For the MIC, after culture for 8 h, the log phase *A. pleuropneumoniae* was diluted to 10^6^ CFU/mL. Then, 100 µL of the bacterial suspension was added to MHA plates containing different DAN concentrations (0.016–1 µg/mL). After drying, the plates were cultured for 18–20 h. The MIC was determined as the minimum drug concentration that prevented bacterial growth.

For the MIC_99_, a series of DAN-containing MHA agar plates were prepared using 10% decreases in the (90%, 80%, 70%, 60%, and 50%). Log phase *A. pleuropneumoniae* was diluted (10^− 1^, 10^− 2^, 10^− 3^, 10^− 4^, 10^− 5^, and 10^− 6^) and dropped onto each MHA plate and cultured for 24 h. The number of *A. pleuropneumoniae* colonies were counted. The ratio of recovery growth was calculated as the number of colonies in each drug-containing plate divided the number on the blank plate and the linear relationship to the drug concentrations was determined. The MIC_99_ was defined as the lowest concentration that inhibited the growth of the bacteria by ≥ 99% (1% recovery).

For MPC, 100 mL of a log phase bacterial suspension was centrifuged at 5000 × *g*, 4 °C for 20 min. The supernatant was removed and 1 mL blank TSB was added to make the bacterial number about 1.5 × 10^11^ CFU/mL. Then, 100 µL of the bacterial suspension was added to a series DAN-containing MHA plates (containing DNA at 1, 2, 4, 8, 16, 32, and 64 MIC) and incubated for 72 h. The minimum concentration of DAN that inhibited bacterial growth was defined as the MPC_pr_. Then, the drug concentration was decreased linearly from 50 to 10% of the MPC_pr_ and the procedures were repeated. MPC was defined as the lowest concentration of DAN that could inhibit the growth of the bacteria. All tests were carried out in triplicate.

### Establishment of the in vitro peristaltic pump model

The peristaltic pump model was constructed according to our previous study [28] and the specific model schematic diagram can found in a published paper [29]. It included a blue-cap bottle (500 − 5000 mL, for storage of fresh media), a three-necked flask (350 mL, representing the central chamber, containing 300 mL of TSB, the dialysis tube (containing 10 mL of bacterial suspension), and magnetic rotor, placed in a large beaker containing water (37 °C) on a thermostatic magnetic stirrer), and a waste liquid collection bottle that was connected through the peristaltic pump and a rubber tube. The PK parameters of DAN in pig lungs were taken from a previously published report [30]. The elimination halflife (t_1/2β_) of DAN in pig lungs was 10.46 ± 0.76 h. For ease of calculation, we set the t_1/2β_ at 12 h. The elimination rate constant (K_el_) was calculated as 0.693/t_1/2β_. The peristaltic pump flow rate (Q) was calculated as K_el_×V_C_ (the central chamber broth volume). After the flow rate was set, the device was run for 2 h to stabilize it. Then, log phase *A. pleuropneumoniae* (10^8^ CFU/mL) was added to the dialysis tube. When the bacterial population was stabilized at about 10^8^ CFU/mL, the in vitro dynamic infection model was successfully established.

### Antibacterial effect and MIC changes during MSW of DAN against *A. pleuropneumoniae*

According to the MIC_99_ and MPC, seven dosing groups were applied: 0, 0.025, 0.05, 0.1, 0.2, 0.4, and 0.8 µg/mL, respectively. To quickly balance the drug concentration in the dialysis tube, at the beginning of the test, the same drug dose was added to the central chamber and the dialysis chamber, simultaneously, three times (24 h/time). Then, 0.1 mL of the bacterial suspension was collected from the dialysis chamber using a 1 mL sterile syringe at 0, 3, 6, 9, 12, and 24 h after each administration, and at 48 and 72 h after the last administration. The agar plate method was applied to determine the bacterial count. Each dose group was assessed three times. The mean value of the bacterial count was applied to draw the dynamic kill curves.

The MIC of DAN toward *A. pleuropneumoniae* was tested at 24 h after each administration and at 48 and 72 h after the last administration. The *A. pleuropneumoniae* with an increased MIC were passaged for five generations to monitor the stability of the MIC.

### PK/PD integration and analysis

The drug concentrations were simulated using a first-order elimination rate process and calculated according to the following formula:

Formula 2 $$C = {C_0} \times {e^{ - kt}}$$

where C is the drug concentrations at time t; C_0_ is the initial concentration of DAN; k is the constant of the elimination rate; and t is the time after drug administration.

The DAN concentrations were calculated and the drug concentration-time curves were plotted. The values of the area under concentration-time curve (AUC_24 h_) and maximum concentration (C_max_) during 24 h were analyzed using a non-compartment model (WinNonlin software).

The antibacterial effect (I) was defined as the maximum change in the number of bacteria during the interval of each administration.

The PK/PD parameters (AUC_24h_/MIC_99_, and C_max_/MIC_99_) were calculated directly using the values of AUC_24h_ and C_max_ divided by the MIC_99_. The percentage of time when the drug concentration was above MIC_99_ during the dosing interval (%T > MIC_99_) was calculated using pharmacodynamic models in the WinNonlin software.

An inhibitory Sigmoid E_max_ model was applied to analyze the relationship between PK/PD parameters and I. The formula was described as follows:

Formula 3 $$I = {I_{\max }} - \frac{{\left( {{I_{\max }} - {I_0}} \right) \times C_e^N}}{{C_e^N + IC_{50}^N}}$$

where I is the bacterial count change at different drug concentrations; I_max_ is the bacterial count change in the control group; I_0_ is the maximum change of bacterial count in the treatment group; C_e_ is the PK/PD parameter; IC_50_ is the value of PK/PD parameter to reach half of the I_0_; and N is the Hill coefficient, used to determine the slope of the PK/PD parameter and I curves.

R^2^ was applied to fit the relation between the PK/PD parameters and I. The bigger the R^2^ value, the better the fitting between PK/PD parameters and I. The values of the PK/PD parameters to produce a bacteriostatic effect (0 Log_10_ CFU/mL reduction), a bactericidal effect (3 Log_10_ CFU/mL reduction), and an eradication effect (4 Log_10_ CFU/mL reduction) were then calculated.

## Results

### MIC and kill curves of DAN against *A. Pleuropneumoniae* in TSB

**Fig. 1:**
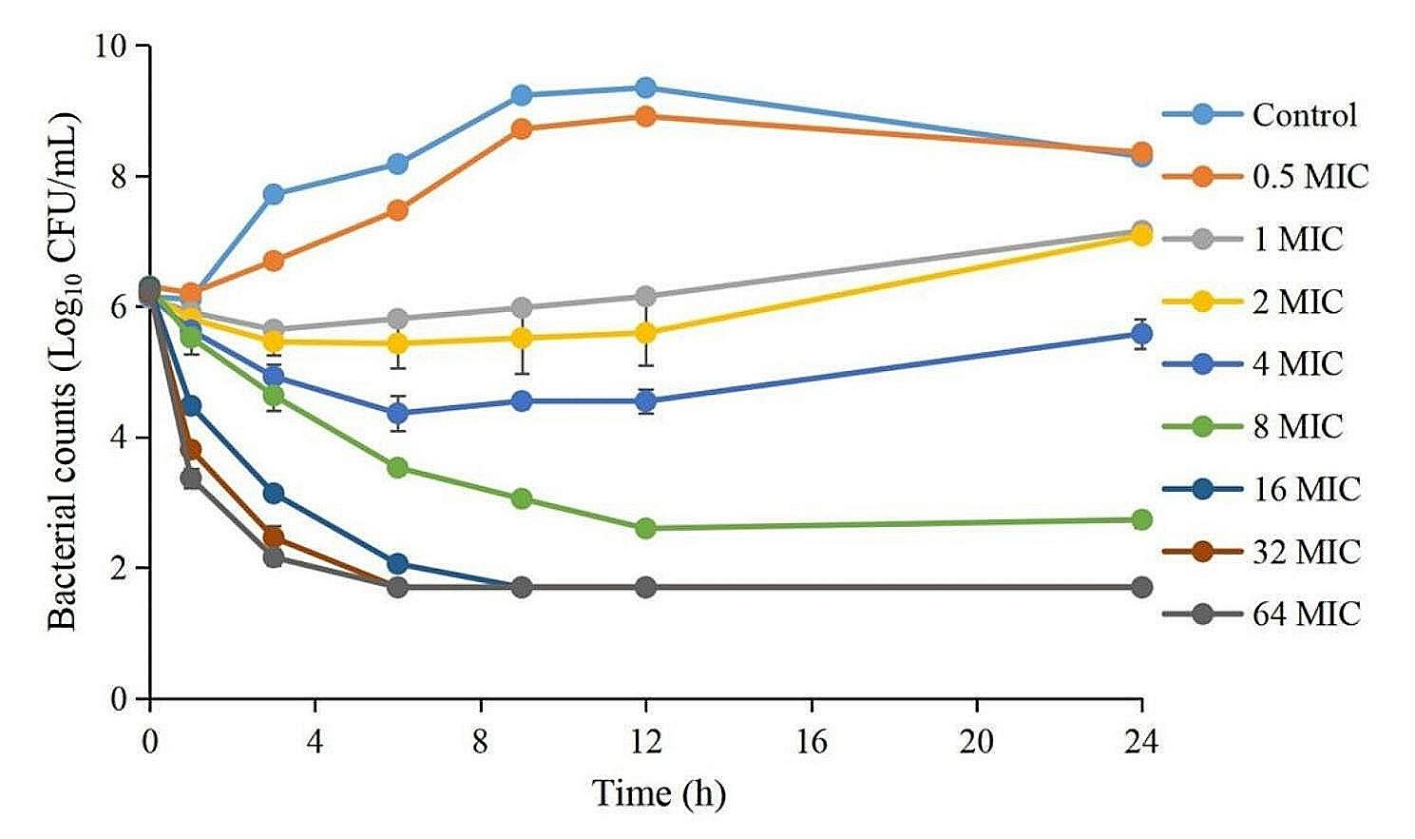
The time-killing curves of danofloxacin against *A. pleuropneumoniae* in TSB at different concentrations. Each symbol represents the mean values ± SD.

The MIC of DAN against *A. pleuropneumoniae* was 0.016 µg/mL in TSB. The change in *A. pleuropneumoniae* numbers in different time periods after being exposed to different DAN concentrations are listed in Table 1. The kill curves are shown in Fig. 1. Table 1 showed that when the DAN concentration was under 4 MIC, a bacteriostatic effect could be produced. When the DAN concentration reached 8 MIC, a bactericidal effect was produced; however, it took a long time (> 6 h). When the concentration of DAN increased to more than 16 MIC, an eradication effect was rapidly produced (< 6 h).

**Table 1:** The population change of *A. pleuropneumoniae* (Log_10_ CFU/mL) at different danofloxacin concentrations in TSB during different time periods

	Concentrations (× MIC)								
Time (h)	0	0.5	1	2	4	8	16	32	64
0 ∼ 1	−0.03	−0.11	−0.22	−0.39	−0.53	−0.79	−1.81	−2.41	−2.89
1 ∼ 3	1.60	0.49	−0.26	−0.35	−0.71	−0.89	−1.34	−1.35	−1.21
3 ∼ 6	0.46	0.78	0.16	−0.03	−0.56	−1.11	−1.08	-	-
6 ∼ 9	1.05	1.24	0.17	0.08	0.19	−0.48	-	-	-
9 ∼ 12	0.12	0.19	0.17	0.08	0.00	−0.45	-	-	-
12 ∼ 24	−1.06	−0.55	1.01	1.49	1.03	0.13	-	-	-
0 ∼ 24	2.15	2.05	−0.48	−0.77	−1.80	−3.71	−6.29	−6.22	−6.26

-: no detected bacteria; 0 ∼ 24: the maximum change of bacterial population during 0–24 h. Values are the mean of triplicated experiments

From the kill curves (Fig. 1), we observed that the growth of *A. pleuropneumoniae* was delayed and did not reach the maximum growth at 0.5 MIC compared with the control group. At 1 − 4 MIC, the number of *A. pleuropneumoniae* gradually decreased with increasing drug concentration, but ultimately recovered. At 8 MIC, DAN produced a bactericidal effect with no bacterial recovery. At 16 − 32 MIC, the number of bacteria decreased rapidly until they were below the limit of detection, and no bacteria recovered.

### PK/PD analysis between the kill rate and DAN concentration

When the drug concentration exceeded 16 MIC, no bacteria could be detected after 6 h. Therefore, in this experiment, we only calculated the kill rate within 3 h and the values during different time periods are listed in Table 2. The results showed that the kill rate increased linearly with increasing DAN concentration during the 0–1 h time period. During 1–3 h, the kill rate also gradually increased under 16 MIC, but did not increase when the concentration exceed 16 MIC. The kill rate in the 0–1 h time period was significantly higher than that in 1–3 h time period, which indicated that DAN produced a rapid antibacterial effect against *A. pleuropneumoniae*.

**Table 2:** Kill rates (Log_10_ CFU/mL/h) of danofloxacin against *A. pleuropneumoniae* under different drug concentrations during different time periods

	Concentrations (× MIC)								
Time (h)	0	0.5	1	2	4	8	16	32	64
0 ∼ 1	0.03	0.11	0.22	0.39	0.53	0.79	1.81	2.41	2.89
1 ∼ 3	−0.80	−0.25	0.13	0.18	0.35	0.44	0.67	0.67	0.61
0 ∼ 3	−0.52	−0.13	0.16	0.25	0.41	0.56	1.05	1.25	1.37

Values are the mean of triplicated experiments

**Fig. 2:**
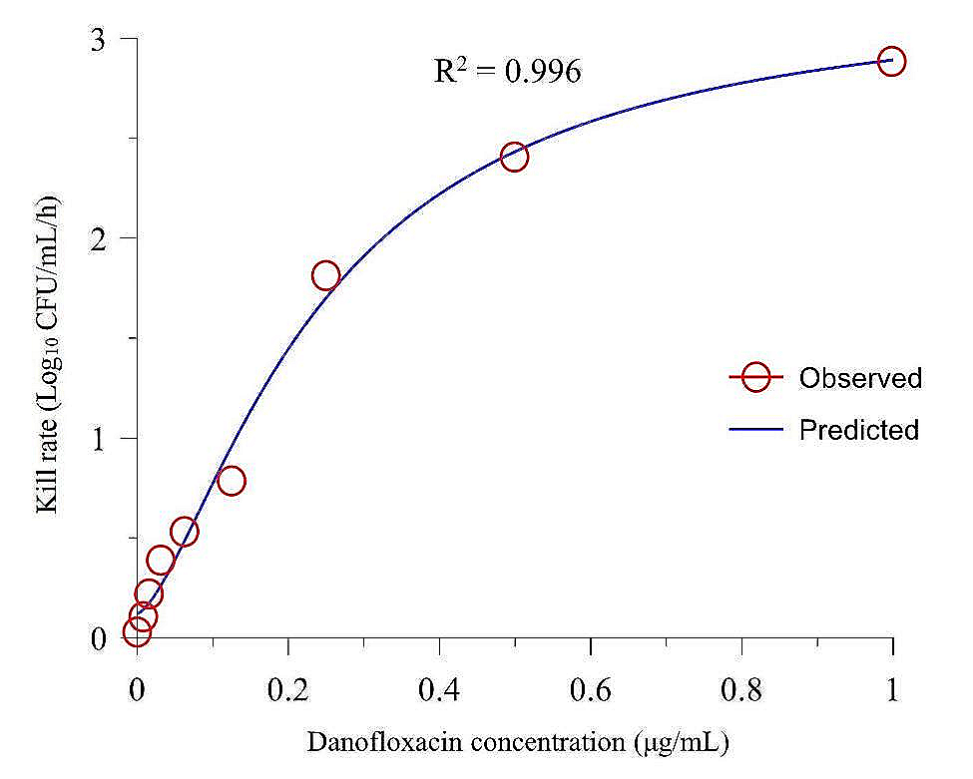
The relationship between danofloxacin concentrations and kill rates over 0–1 h after Sigmoid E_max_ simulation. The R^2^ value isthe degree of fit between the kill rate and the drug concentration

The PK/PD parameters between the kill rate and DAN concentrations during each time period are listed in Table 3. The results showed that the correlation between the kill rate and DAN concentration was very high at different time periods (the R^2^ values range from 0.991 to 0.996). The time period with the best fit correlation with the drug concentration was 0–1 h (Fig. 2), and its maximum kill rate was 3.23 Log_10_ CFU/mL/h. Figure 2 shows the fitted curve between the kill rate and DAN concentrations for the 0–1 h time period, showing that the kill rate correlated positively with the drug concentration.

**Table 3:** The main PK/PD parameters between danofloxacin concentrations and kill rates according to Sigmoid E_max_ simulation

Time (h)	E_max_ (Log_10_ CFU/mL/h)	EC_50_ (µg/mL)	E_0_ (Log_10_ CFU/mL/h)	N	R^2^
0 ∼ 1	3.23	0.25	0.12	1.49	0.996
1 ∼ 3	0.70	0.01	−0.80	0.81	0.991
0 ∼ 3	2.29	0.21	−0.52	0.52	0.992

E_max_ is the maximum kill rate of DAN during each period; E_0_ the kill rate in the blank TSB control; C_e_ the DAN concentration; N the Hill coefficient, representing the steepness of the curve between the kill rate and the DAN concentration; EC_50_ is the DAN concentration that produces a 50% maximum kill rate; R^2^ is the degree of fit between the kill rate and the drug concentration

### MIC, MIC_99_, and MPC in MHA

The MIC, MIC_99_ and MPC values of DAN against *A. pleuropneumoniae* were 0.0625, 0.05, and 0.4 µg/mL in MHA, respectively.

### PK and in vitro dynamic kill curves

**Fig. 3:**
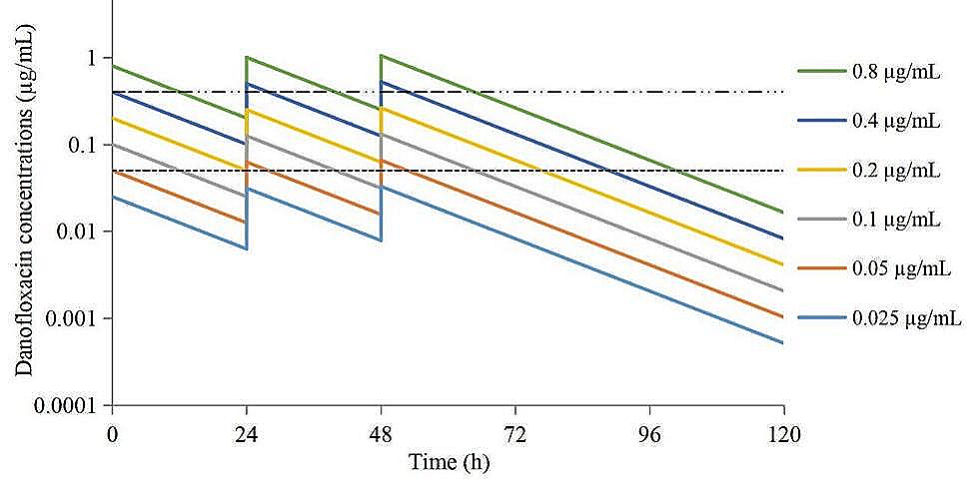
The simulated concentration-times curves of danofloxacin in the peristaltic pump model. The dotted line represents the MPC (upper), the dashed line represents the MIC_99_ (lower)

According to formula 2, the drug concentrations of DAN at each time point were obtained by extrapolation. After WinNonlin analysis, the C_max_ and AUC_24h_ were obtained after each administration. The semi-logarithmic concentration-time curves are shown in Fig. 3. The results showed that the drug concentration was located in different parts of the MSW. The 0.025 µg/mL group was located outside the MSW, the 0.05 and 0.1 µg/mL groups were partially located in the lower part of the MSW, and the 0.2 and 0.4 µg/mL groups were located in the middle part of the MSW. The 0.8 µg/mL group was located in the upper part and outside the MSW.

**Fig. 4:**
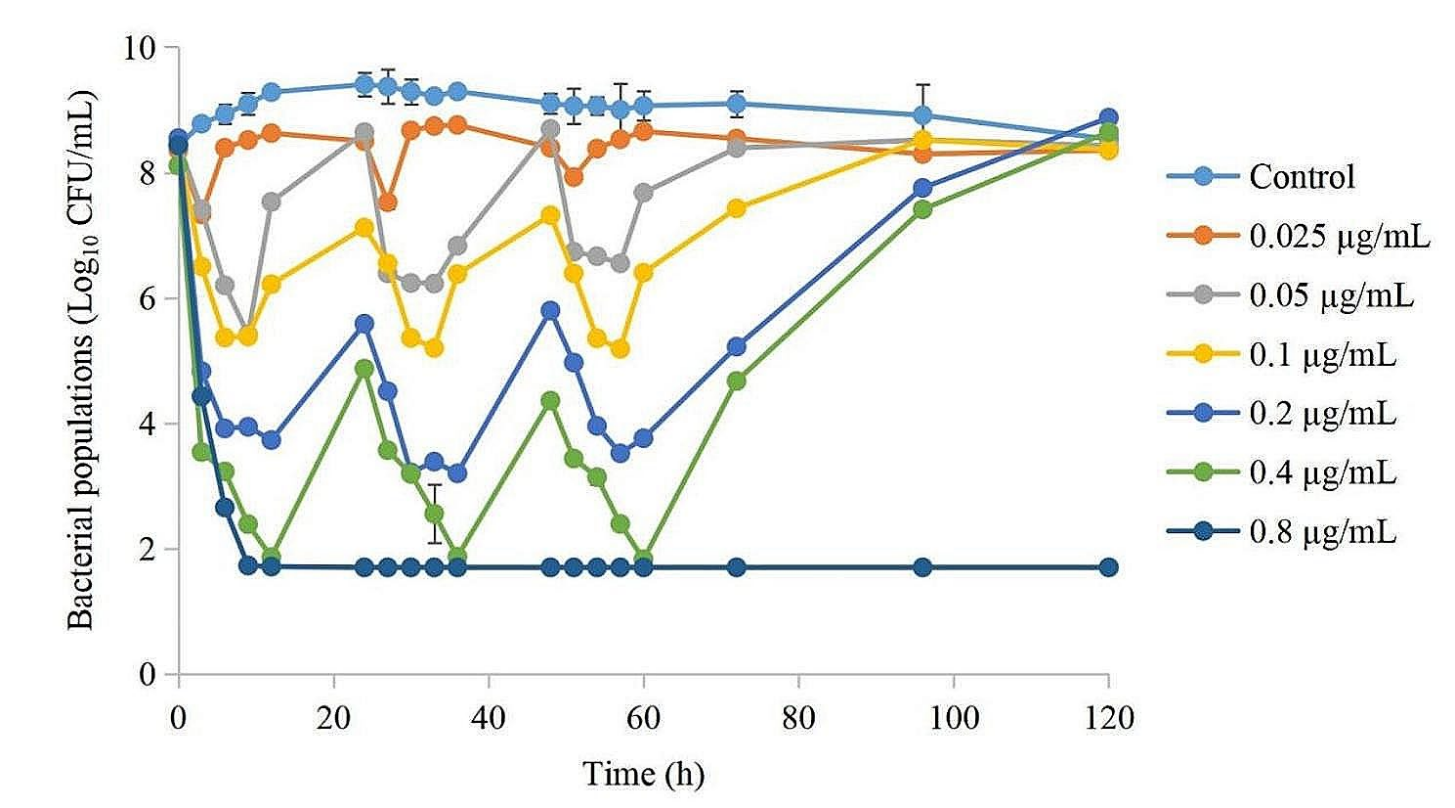
The time kill curves of danofloxacin against *A. pleuropneumoniae* at different drug concentrations in the peristaltic pump model

The kill curves under different DAN concentrations are shown in Fig. 4. The results showed that all the bacterial populations decreased, but eventually recovered after each administration (except for 0.8 µg/mL). The antibacterial effects are shown in Table 4. The 0.025 µg/mL group could achieve a bacteriostatic effect, the 0.05 and 0.1 µg/mL groups could achieve a bactericidal effect, and the 0.2–0.8 µg/mL groups could achieve an elimination effect. We can also observed that the antibacterial effect after each administration has little different in the 0.025 µg/mL group. However, when the drug concentration exceed 0.025 µg/mL, the bacterial reduction after the first administration was significantly higher than that after the second and third administrations. The greater the dose, the greater the difference, while the difference between the second and third administrations is not significant.

**Fig. 5:**
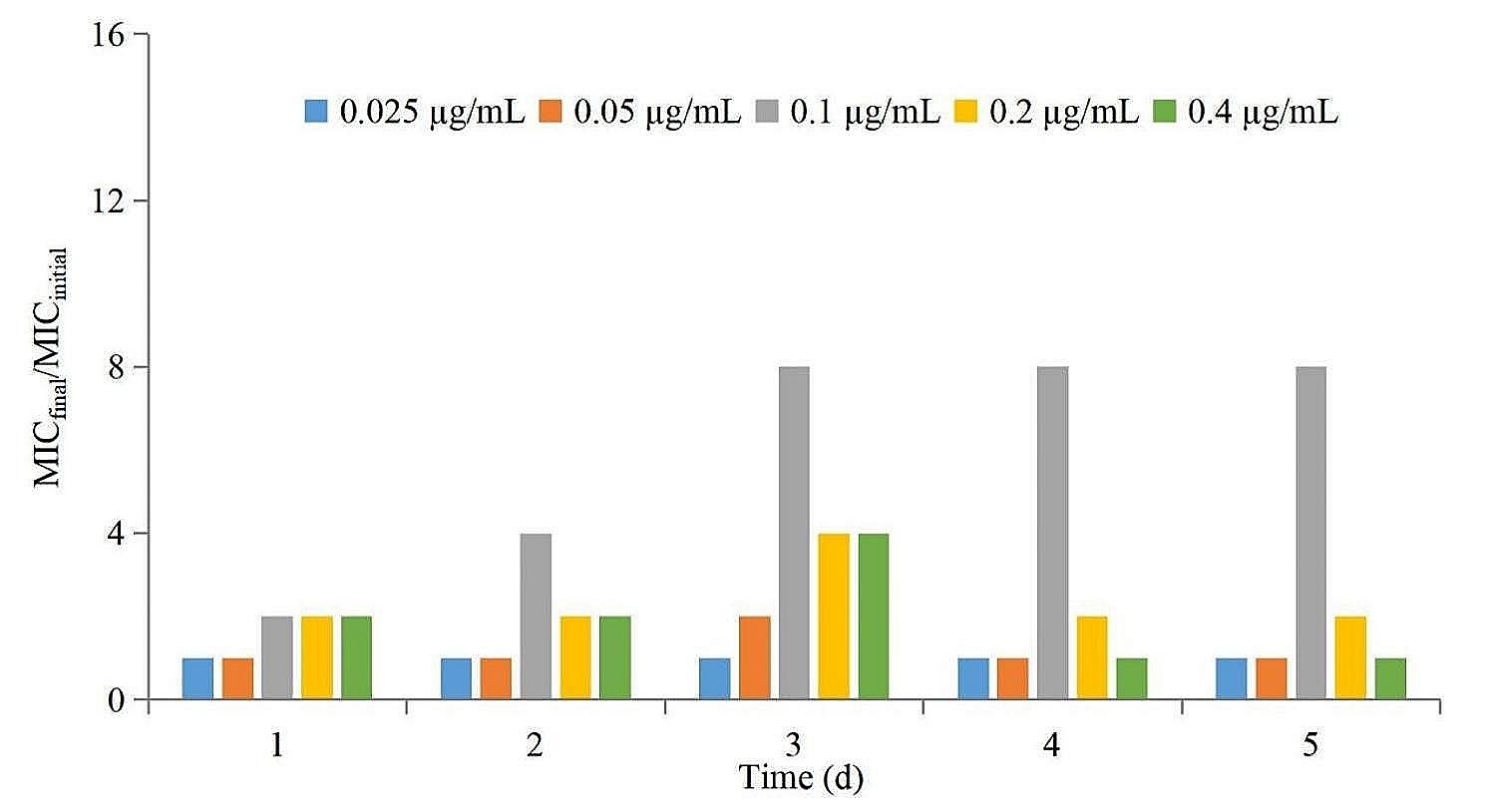
The values of MIC_final_/MIC_initial_ ratio after drug administration each time point

The MIC changes of *A. pleuropneumoniae* under each dosage are depicted in Fig. 5. The results showed that when the drug concentration was lower than the MIC_99_ and higher than the MPC, the MIC of *A. pleuropneumoniae* did not change. When the drug concentrations were in the middle of the MSW, the MIC would increase significantly with increasing administration times (increased by 8-fold in the 0.1 µg/mL group, and by 2-fold in the 0.2 µg/mL group), and would recover to the initial value after the third administration (in the 0.05 and 0.4 µg/mL groups).

**Table 4:** The antibacterial effect (I) values and PK/PD parameters of danofloxacin against *A. pleuropneumoniae* during different administration intervals

Doses	AUC_24h_/MIC_99_ (h)	C_max_/MIC_99_	%T > MIC_99_ (%)	I (Log_10_ CFU/mL)
Control	0	0	0	0.98
	0	0	0	−0.3
	0	0	0	−0.1
0.025	6.59	0.50	0	−1.02
	8.24	0.63	0	−0.98
	8.65	0.66	0	−0.46
0.05	13.18	1.00	0	−3.06
	16.47	1.25	16.32	−2.41
	17.30	1.31	19.88	−2.14
0.1	26.35	2.00	50.00	−3.07
	32.94	2.50	70.00	−1.92
	34.59	2.63	73.81	−2.13
0.2	52.70	4.00	100.00	−4.82
	65.89	5.00	100.00	−2.39
	69.18	5.25	100.00	−2.04
0.4	105.41	8.00	100.00	−6.25
	131.78	10.00	100.00	−3.00
	138.37	10.50	100.00	−2.53
0.8	210.82	16.00	100.00	−6.73
	263.55	20.00	100.00	-
	276.74	21.00	100.00	-

AUC_24 h_, 24-h area under concentration-time curve; C_max_, maximum concentration; MIC_99_, the minimum concentration that inhibits colony formation by 99%; %T > MIC_99_, the percentage of time that drug concentration remained above MIC_99_; Each dose was administered three times. Each dose group was created and analyzed in triplicate

### PK/PD integration

**Fig. 6:**
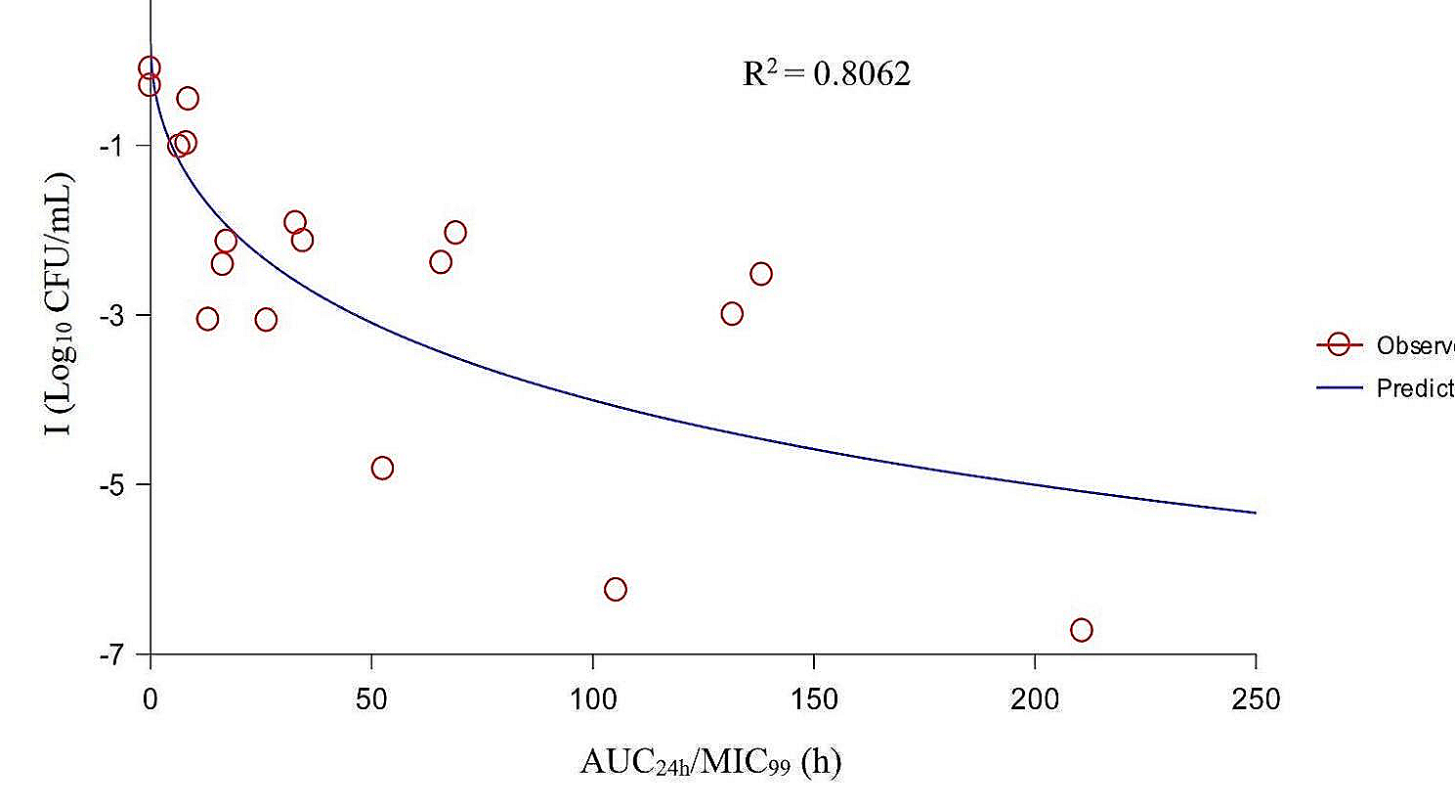
The fitting curve between AUC_24h_/MIC_99_ and the antibacterial effect

**Fig. 7:**
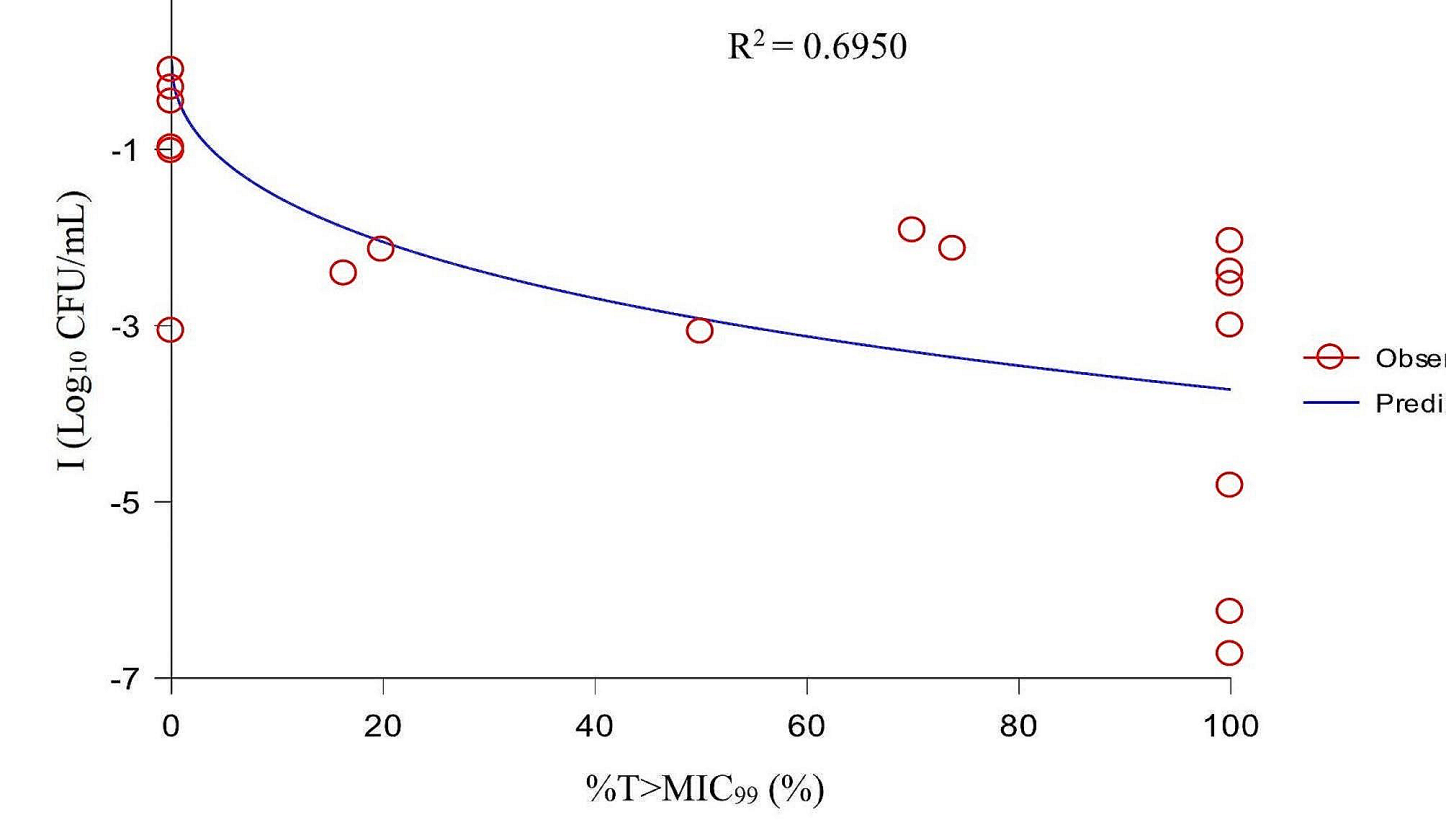
The fitting curve between %T > MIC_99_ and the antibacterial effect

**Fig. 8:**
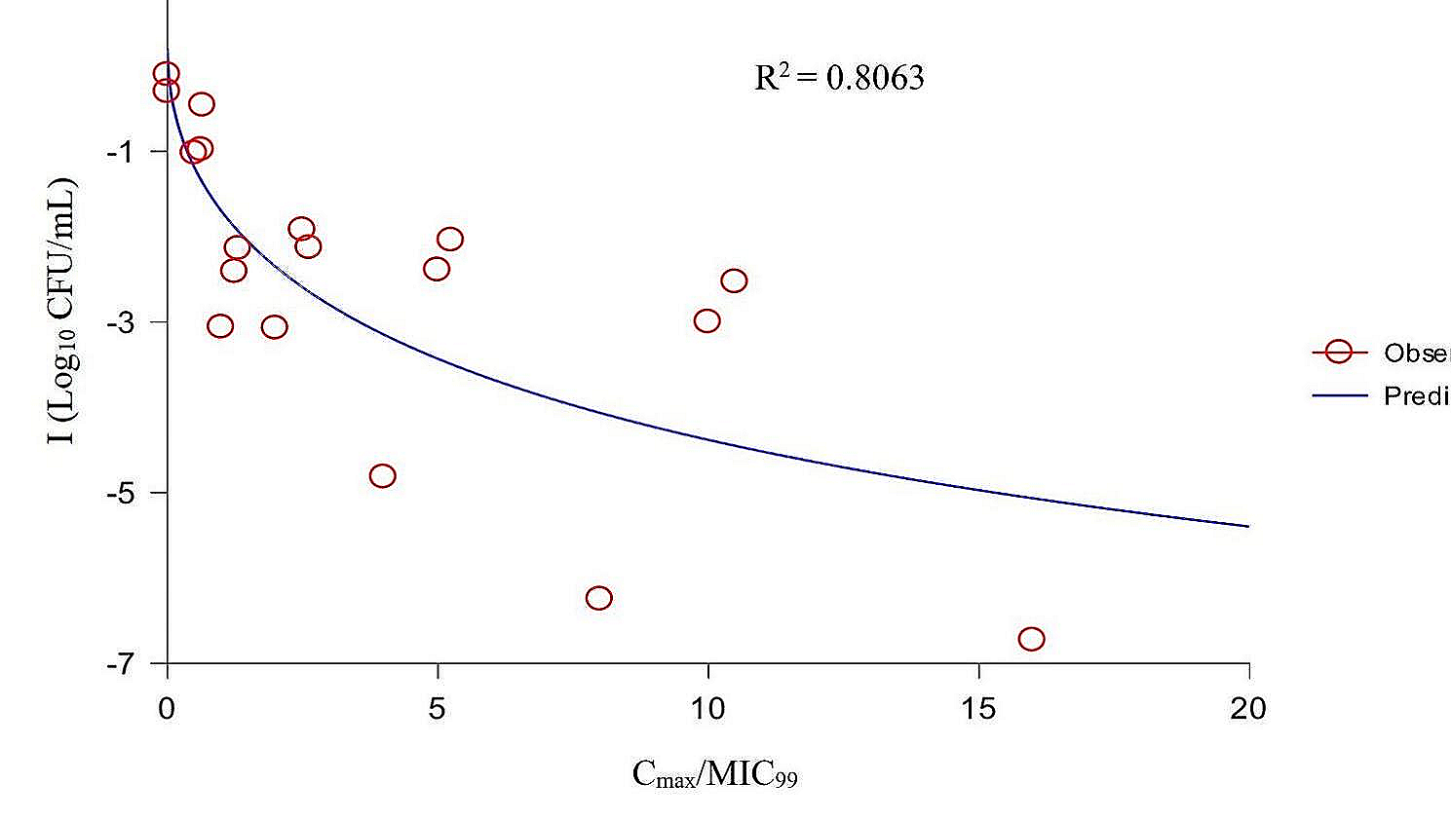
The fitting curve between C_max_/MIC_99_ and the antibacterial effect

The values of PK/PD parameters and the corresponding I values are shown in Table 4. After PK/PD integration, the relationships between PK/PD parameters and I were exhibited in Figs. 6 and 7, and 8.

Based on the R^2^ values, we selected AUC_24h_/MIC_99_ as the PK/PD parameter for analysis. The values of the PK/PD parameters and AUC_24h_/MIC_99_ required to predict different antibacterial effects are shown in Table 5. The results showed that the AUC_24h_/MIC_99_ values required to achieve bacteriostatic, bactericidal, and eradication effects were 9.46, 25.14, and 36.06 h, respectively.

**Table 5:** Values of the PK/PD parameters and AUC_24h_/MIC_99_ required to achieve different antibacterial effects

PK/PD parameters	Values
I_max_ (Log_10_ CFU/mL)	0.19
IC_50_ (h)	257.66
I_0_ (Log_10_ CFU/mL)	−10.95
Slope (N)	0.53
AUC_24h_/MIC_99_ for bacteriostatic effect (h)	9.46
AUC_24h_/MIC_99_ for bactericidal effect (h)	25.14
AUC_24h_/MIC_99_ for eradication effect (h)	36.06

I_max_: change in the bacterial count in the control group after administration of each dose; I_0_: maximum change in the bacterial count in the treatment group after administration of each dose; IC_50_: value of the PK/PD parameter to reach half of I_max_; N: Hill coefficient, representing the slope of the PK/PD parameter and E curves

## Discussion

*A. pleuropneumoniae* is a pathogen that can cause serious respiratory diseases in pigs. Antibiotics have played an important role in treating infectious diseases. However, with the extensive use of antibiotics, bacteria have gradually developed drug resistance [31–33]. New drugs could effectively treat drug resistant bacteria; however, the speed of drug development cannot keep pace with the increase in drug-resistant bacteria. Therefore, optimizing dosage regimens of currently available drugs is a more realistic method.

The static time kill curve (STKC) is a basic method to study the antibacterial characteristics of drugs, which can be used to intuitively understand the antibacterial activity of drugs against pathogens by determining the kill rate, antibacterial effect, and bacterial regrowth [34]. The kill rate is a pharmacodynamic parameter obtained based on MIC and kill curves, which represents the slope of the kill curve at a certain time period, and is the combined result of the bacterial growth rate and death rate, thereby reflecting the dynamic relationship between the kill rate and drug concentrations. The kill rate method directly reflects the antibacterial activity of the drug itself and can divide drugs into concentration-dependent and time-dependent drugs [28]. The PK/PD integration between the kill rate and drug concentrations in different time periods can describe the bactericidal kinetic characteristics of drugs against bacteria in more detail [35, 36].

In this study, DAN exhibited concentration-dependent antibacterial effects against *A. pleuropneumoniae* based on the kill rate. With the increase in drug concentration, the bacterial population decreased rapidly. When the drug concentration increased to 64 MIC, only 3 h was needed to achieve an eradication effect. These results are consistent with those of other researchers. Tomc et al. [37] studied the kill rate of fluoroquinolones against different bacteria, showing that fluoroquinolones were more effective than βlactam drugs, and the times required to produce a bactericidal effect were 1.5 h for *Enterobacteriaceae*, 4–6 h for *Staphylococcus aureus*, and ≥ 6 h for *Streptococcus*. The results of the kill rate at 1–3 h showed that the kill rate did not continue to increase with the increase of the drug concentration. One reason could be that when the drug concentration increased to 16 MIC, the number of bacteria decreased rapidly and soon fell below the detection limit, which affected the calculation of the kill rate.

For *A. pleuropneumoniae*, the target infectious organ is the lung, making it difficult to obtain real-time and continuous dynamic PK and PD data in pigs. To date, a tissue cage model [12] has been applied for PK/PD analysis of DAN against *A. pleuropneumoniae*. However, such studies cannot truly reflect the antibacterial effect in clinical infection, because the drug PK values are different between tissue fluid and the lung. The peristaltic pump model can simulate the dynamic change in drug concentrations and bacterial populations in the target organs, which can reflect a realtime antibacterial effect, especially when simulating the PK and PD in difficult to obtain organs. Therefore, in this experiment, a peristaltic pump infection model was used to carry out the MSW study of DAN against *A. pleuropneumoniae* by simulating the PK values for DAN in pig lungs.

From the MSW study, we found that the antibacterial effect was different after each drug administration. The antibacterial effect after the first administration was significantly higher than that after the second and third treatments. There are several possible explanations for this phenomenon. Firstly, the initial bacterial population (10^8^ CFU/mL) is higher than that in other studies (10^6^ CFU/mL), which might have resulted in the population of sensitive bacteria being relatively high. After the first drug administration, a high proportion of the *A. pleuropneumoniae* population could be inhibited or killed, which could produce a greater antibacterial effect (a large change in the bacterial population). When the drug concentration decreased, it still could inhibit bacterial growth, resulting in the *A. pleuropneumoniae* population being unable to recover to the maximum value. After the second administration, the remaining initial bacterial population was small, resulting in a smaller antibacterial effect. Secondly, after a large number of sensitive bacteria were killed, the insensitive bacteria were screened out. However, the growth rate of the insensitive bacteria might be slower than that of the sensitive bacteria. Therefore, the total amount of bacteria could not recover to the initial level when the drug concentration declined, which could affect the calculation of the antibacterial effect.

In this experiment, we found that the MIC of *A. pleuropneumoniae* increased when the DAN concentrations were located between the MIC_99_ and the MPC. These results were consistent with previous reports [38–40]. There are two reasons for this phenomenon. Firstly, in the original flora, sensitive bacteria are the dominant bacteria, whereas there are fewer insensitive drug-resistant bacterial subpopulations. When the drug concentration was between MIC_99_ and MPC, the sensitive bacteria were gradually killed after multiple administrations and the insensitive bacteria gradually increased and became the dominant flora. Another reason could be that sensitive bacteria and insensitive bacteria had gene mutations under continuous drug selection pressure. *A. pleuropneumoniae* might have multiple drug-resistant gene mutations, which could make it more resistant to DAN. In this study, we found the MIC was increased by 8fold in 0.1 µg/mL group, and by 2-fold in the 0.2 µg/mL group (the corresponding mean values of AUC_24h_/MIC_99_ were 31.29 and 62.59 h, respectively), which might produce multiple genetic mutations in *A. pleuropneumoniae*.

When selecting drugs to treat bacterial infections, PK/PD parameters are an important reference index [41]. For fluoroquinolones, AUC_24h_/MIC is the most commonly applied PK/PD parameter [42]. To date, several studies have reported the PK/PD integration of DAN against ruminant pathogenic bacteria in ex vivo. One study [43] established a sheep tissue cage model to study the PK/PD of DAN against *Mantella hemolytica* in serum and tissue cage fluid, in which the values of AUC_24h_/MIC required to achieve bacteriostatic, bactericidal, and elimination effects were 17.8, 20.2, and 28.7 h in serum and 20.6, 25.5, and 41.6 h, in tissue cage fluid, respectively. Shojaee et al. [44] established a cattle tissue cage model to study the PK/PD of DAN against *M. haemolyticus* in serum and tissue cage fluid, and the values of AUC_24h_/MIC to produce bacteriostatic, 50% population reduction, bactericidal, and elimination effects were 15.9, 16.7, 18.15, and 33.5 h in serum, and 15.0, 16.34, 17.8, and 30.7 h in tissue cage fluid, respectively. There have been reports of ex vivo PK/PD analysis of DAN against porcine pathogenic bacteria. Li et al. [45] studied the PK/PD integration of DAN against *Pasteurella multocide* and *Haemophilus parasuis* in piglet serum, and the results showed that the mean AUC_24h_/MIC values for bacteriostatic and bactericidal effects were 32 and 49.8 h for *P. multocide*, and 14.6 and 37.8 h for *H. parasuis*, respectively. Yang et al. [46] used an ultrafiltration probe model to study the PK/PD integration of DAN against *Escherichia coli* in piglet ileal samples, and the mean values of AUC_24h_/MIC for bacteriostatic, bactericidal, and eradication effects were 99.85, 155.57, and 218.02 h, respectively. In the present study, the results showed that the correlation of AUC_24h_/MIC_99_ and C_max_/MIC_99_ (the R^2^ values were 0.8062 and 0.8063, respectively) were better than that of %T > MIC_99_ (R^2^ = 0.6950) to I. Considering that AUC_24h_ also had the property of timeliness, we used AUC_24h_/MIC_99_ for PK/PD integration. The values of AUC_24h_/MIC_99_ to achieve bacteriostatic, bactericidal, and elimination effects were 9.46, 25.14, and 36.06 h, respectively. However, a multiple gene mutant resistant *A. pleuropneumoniae* might emerge when the AUC_24h_/MIC_99_ is located between 31.29 and 62.59 h; therefore, the value of AUC_24h_/MIC_99_ required to produce elimination effects should be greater than 62.59 h.

## Conclusions

In conclusion, DAN exhibited a concentration-dependent antibacterial activity against *A. pleuropneumoniae* according to the kill rate. The maximum value of kill rate was 3.23 Log_10_ CFU/mL/h during the 0–1 h period. When the drug concentration was located in the middle part of the MSW, drug-resistant bacteria might be induced. Therefore, this dosage should be avoided to produce a mean value of AUC_24h_/MIC_99_ between 31.29 and 62.59 h. The values of AUC_24h_/MIC_99_ to achieve bacteriostatic, bactericidal, and eradication effects were 9.46, 25.14, and > 62.59 h, respectively. We believe these kill rates and MSW results will provide a valuable guidance for the use of DAN to treat *A. pleuropneumoniae* infections.

## Data Availability

The data used in the study analyses can be made available by the corresponding author on reasonable request.

## Abbreviations

MSW: mutant selection window
MIC: minimum inhibitory concentration
TSB: tryptic soy broth
MPC: mutant prevention concentration
PK/PD: pharmacokinetic/pharmacodynamic
TCF: tissue cage fluid
MHA: Mueller-Hinton agar
NAD: Nicotinamide adenine dinucleotide
CFU: Colony forming units
